# Infusion Therapies in the Treatment of Parkinson’s Disease

**DOI:** 10.3233/JPD-225112

**Published:** 2023-07-25

**Authors:** Teus van Laar, K. Ray Chaudhuri, Angelo Antonini, Tove Henriksen, Maja Trošt

**Affiliations:** a Department of Neurology, University Medical Center Groningen, University of Groningen, The Netherlands; b Parkinson’s Foundation International Centre of Excellence, King’s College Hospital, London, UK; c Psychology & Neuroscience, King’s College Institute of Psychiatry, London, UK; d Parkinson and Movement Disorders Unit, Study Center on Neurodegeneration (CESNE), Department of Neuroscience, Padua University, Padua, Italy; e Department of Neurology, Movement Disorder Clinic, University Hospital of Bispebjerg, Copenhagen, Denmark; f Department of Neurology, University Medical Centre Ljubljana, Slovenia, Faculty of Medicine, University of Ljubljana, Slovenia

**Keywords:** Continuous subcutaneous infusion, apomorphine, carbidopa, levodopa drug combination, implantable infusion pumps, Parkinson’s disease

## Abstract

Oral levodopa is the gold-standard therapy for treating Parkinson’s disease (PD) but after a few years of treatment the therapeutic window narrows, and patients often experience various treatment-related complications. Patients in this advanced PD stage may benefit from alternative therapy, such as continuous intrajejunal delivery of levodopa-carbidopa intestinal gel (LCIG; or carbidopa-levodopa enteral suspension), continuous intrajejunal delivery of levodopa-carbidopa-entacapone intestinal gel, or continuous subcutaneous apomorphine infusion. Consideration and initiation of infusion therapies in advanced PD are suggested before the onset of major disability. The present review summarizes clinical evidence for infusion therapy in advanced PD management, discusses available screening tools for advanced PD, and provides considerations around optimal use of infusion therapy.

## INTRODUCTION

Levodopa is the most effective therapy in treating Parkinson’s disease (PD) [1–3]. However, as the disease progresses, loss of striatal dopamine nerve terminals and diminished dopamine buffering capacity occurs [2–5]. Delayed gastric emptying associated with dysautonomia may also alter plasma levodopa bioavailability and contribute to an unpredictable response to oral therapy [6–8]. As a result, motor and/or non-motor fluctuations can occur in which patients alternate between good clinical efficacy (“On” time) and inadequate clinical efficacy (“Off” time) [3]. Levodopa-related fluctuations in PD are associated with non-physiological, discontinuous, or pulsatile stimulation of striatal dopamine receptors and can lead to unwanted motor symptoms known as dyskinesias [3, 9].

Dopamine agonists, catechol-O-methyltransferase inhibitors, and monoamine oxidase-B inhibitors can be added to treatment regimens to prolong oral levodopa efficacy, reduce “Off” time, and improve motor disability [1, 6]. However, polypharmacy and numerous doses may result in reduced treatment adherence [10] and additional side effects [1, 3, 11].

Infusion and surgical therapies offer an alternative to oral medication for patients with advanced PD (aPD) if symptoms are inadequately controlled [12–15], and recent European Academy of Neurology/Movement Disorder Society guidelines indicate alternative therapies can be considered for aPD with fluctuations not sufficiently managed with oral treatments [16]. Invasive surgical therapy, such as deep brain stimulation is an alternative intervention that has been extensively studied in patients with aPD with fluctuations and is recommended as a potential therapeutic option for patients with aPD [16]; however, deep brain stimulation is outside of the scope of this review. The present review provides an up-to-date overview of the efficacy of infusion therapies in patients with aPD, focuses on tools to identify patients who may benefit from these therapies, and provides considerations around optimal use of these therapies.

## OVERVIEW OF EXISTING INFUSION THERAPIES

### LCIG/CLES

Continuous dopaminergic stimulation using levodopa-carbidopa intestinal gel (LCIG; also known as carbidopa-levodopa enteral suspension [CLES]) is approved internationally by multiple regulatory authorities and is indicated for 16-hour daily infusion of levodopa-carbidopa in patients with aPD [17, 18]. If medically justified, it can be used up to 24 hours in certain countries. LCIG/CLES is designed to limit pulsatile stimulation due to erratic gastric emptying by continuously administering levodopa-carbidopa directly into the duodenum or upper jejunum from a portable pump via a percutaneous endoscopic gastrostomy with a jejunal extension tube [17, 19].

Clinical and real-world evidence of LCIG/CLES safety and efficacy in patients with aPD (Tables 1–4) is available to help inform clinical decision-making [20–25]. The first randomized, controlled, crossover multicenter study to investigate the efficacy of LCIG/CLES infusion assessed 24 patients with aPD whose motor symptoms were not well controlled by their current regimen [26]. Compared to individualized conventional treatment, LCIG/CLES treatment resulted in better scores on the treatment response scale, which measured the “On” and “Off” states (*p* <  0.01), total Unified Parkinson’s Disease Rating Scale (UPDRS) scores (*p* <  0.05), lower median UPDRS total and Parts I, II, and IV scores, and significantly *(p* <  0.01) improved nearly all quality of life dimensions on the 39-item Parkinson’s Disease Questionnaire and the generic quality of life (QoL) instrument 15D [26]. A subsequent randomized controlled trial investigating the efficacy of LCIG/CLES in improving motor symptoms showed that 16-hour continuous infusion with LCIG/CLES significantly improved “Off” time (*p* = 0.0015), “On” time without dyskinesia (*p* = 0.0142), and “On” time without troublesome dyskinesia (*p* = 0.0059) (Table 2) [20]. Continuous infusion with LCIG/CLES over 12 weeks also improved patients’ QoL and activities of daily living (ADL) (Table 3) [20]. Similar results were reported in DYSCOVER, a phase 3b, open-label, multicenter, 12-week, interventional study in 63 patients with aPD whereby dyskinesias, “On” time without troublesome dyskinesia (*p* = 0.0001), QoL, global impression of change (both *p* <  0.0001) and activities of daily living (*p* = 0.0006) improved with LCIG/CLES vs. optimized medical treatment [23].

**Table 1:** Patient characteristics and demographics at studies’ baseline

	LCIG/CLES							CSAI			
Variable	Olanow et al., 2014 (NCT00357994 and NCT00660387) [20]	GLORIA [21]	COSMOS (NCT03362879) [22]	GREENFIELD [24]	DYSCOVER (NCT02799381) [23]	DUOGLOBE^a^ (NCT02611713) [25]	EuroInf (LCIG/CLES) [44]	TOLEDO (NCT02006121) [40]	EuroInf (CSAI) [44]	Borgemeester et al., 2016 [46]	Sesar et al., 2017 [47]
N	37	375	409	145	28	195	44	53	43	125	230
Mean (SD) age, y	63.7 (9.5)	66.4 (8.8)	66.5 (7.8)	70.4 (7.7)	69.3 (7.0)	70.2 (8.2)	62.7 (9.1)	63.6 (9.3)^b^	62.3 (10.6)	65.8 (9.8)	67.3 (8.3)
Sex, %
Male	64.9	58.7	65.3	50.3	42.9	61.5	56.8	64.0	48.8	61.6	NA
Female	35.1	41.3	34.7	49.7	57.1	38.5	43.2	36.0	51.2	38.4	NA
White, %	94.6	NA	NA	NA	NA	NA	NA	NA	NA	NA	NA
BMI, kg/m^2^, mean (SD)	NA	NA	NA	NA	24.5 (4.2)	25.9 (4.1)	NA	NA	NA	NA	NA
Mean age at diagnosis, y	NA	NA	NA	55.7 (9.3)	NA	NA	NA	NA	NA	NA	NA
Mean (SD) time from diagnosis, y	10.0 (4.6)	12.7 (6.3)	12.8 (5.4)	14.6 (6.6)^c^	12.7 (4.2)	11.2 (4.8)	16.1 (6.7)	11.8 (5.6)	14 (4.5)	11.9 (5.7)	14.2 (6.3)
“Off” time, UPDRS IV item 39, mean (SD) hours/day	NA	6.0 (3.2)	NA	2.0 (0.8)	NA	6.0 (3.4)	NA	NA	NA	NA	NA
“Off” time, patients’ diary, mean (SD) hours/day	6.3 (1.7)	NA	6.1 (3.6)	NA	4.8 (2.4)	6.0 (3.4) (patient report)	NA	6.7 (2.2)	NA	NA	NA
Dyskinesia duration, UPDRS IV item 32, mean (SD) hours/day	NA	4.3 (3.8)	NA	1.8 (1.0)	NA	NA	NA	NA	NA	NA	NA
“On” time with troublesome dyskinesia, patient’s diary, mean (SD) hours/day	1.0 (1.6)	NA	3.7 (3.4)	NA	2.4 (1.8)	NA	NA	NA	NA	NA	NA
“On” time without troublesome dyskinesia, patient’s diary, mean (SD) hours/day	8.7 (2.0)	NA	—	NA	8.8 (2.9)	NA	NA	8.5 (2.4)	NA	NA	NA
UPDRS II, mean (SD)	11.6 (6.9)	16.5 (9.8)	16.6 (7.8)	29.2 (9.6)^d^	18.3 (6.4)	14.8 (7.8)	NA	NA	NA	NA	NA
UPDRS III, mean (SD)	18.1 (9.9)	24.6 (12.0)	30.1 (15.3)	NA	26.3 (6.7)	27.6 (13.2)	27.3 (12.3)	NA	30.8 (10.4)	NA	NA
UPDRS IV, mean (SD)	NA	NA	NA	NA	NA	NA	9.9 (3.3)	NA	10.0 (4.7)	NA	NA
MMSE total score, mean (SD)	28.7 (1.4)	NA	27.5 (2.8)	NA	28.1 (1.7)	27.7 (2.2)	NA	NA	NA	NA	NA
UDysRS total score, mean (SD)	NA	NA	NA	NA	53.2 (12.2)	33.7 (21.1)	NA	NA	NA	NA	NA
NMSS total score, mean (SD)	NA	69.2 (42.1)	NA	NA	NA	87.9 (51.3)	91.0 (45.0)	NA	82.4 (49.5)	NA	NA
PDSS-2 total score, mean (SD)	NA	NA	NA	25.0 (10.4)	NA	26.6 (11.7)	NA	NA	NA	NA	NA
GFQ, mean (SD)	NA	NA	NA	29.7 (13.3)	NA	NA	NA	NA	NA	NA	NA
QUIP-RS, mean (SD)	NA	NA	NA	10.4 (16.6)	NA	NA	NA	NA	NA	NA	NA
MCSI, mean (SD)	NA	NA	NA	NA	NA	10.9 (6.4)	NA	NA	NA	NA	NA
PDQ-39, mean (SD)	35.1 (18.0)	NA	NA	72.3 (23.8)	NA	NA	NA	NA	NA	NA	NA
PDQ-8, mean (SD)	NA	46.8 (18.6)	NA	NA	45.1 (20.5)	45.1 (18.1)	48.6 (14.6)	NA	49.9 (16.6)	NA	NA
EQ-5D, mean (SD)	NA	0.4 (0.3)	NA	NA	NA	NA	NA	NA	NA	NA	NA
EQ -VAS, mean (SD)	NA	48.0 (21.3)	NA	NA	NA	NA	NA	NA	NA	NA	NA
PD Medications, %					
Levodopa	100	97.9	75.6	96.6	NA	NA	NA^e^	100	30.2	NA	NA
DA agonists	59	67.5	34.0	64.1	NA	NA	9.1	91.0	48.8	NA	NA
COMT inhibitors	49	56.5	13.7	44.1	NA	NA	NA^e^	60.0	30.2	NA	NA
MAO-B inhibitors	41	35.5	18.1	14.5	NA	NA	NA	43.0	NA	NA	NA
Amantadine	NA	27.2	NA	17.2	NA	NA	9.1	30.0	NA	NA	NA
NMDA antagonists	NA	NA	12.5	NA	NA	NA	NA	NA	NA	NA	NA
Apomorphine injection	NA	NA	1.7	9.7	NA	NA	NA	NA	NA	NA	NA
Apomorphine Cl	NA	NA	0.7	4.8	NA	NA	NA	NA	NA	NA	NA
Anticholinergics	NA	NA	2.2	NA	NA	NA	NA	NA	NA	NA	NA
Other oral	NA	13.3	1.5	NA	NA	NA	NA	NA	NA	NA	NA

This review does not include all studies published for each infusion therapy. BMI, body mass index; Cl, chloride; CLES, carbidopa-levodopa enteral suspension; COMT, catechol-O-methyltransferase; COSMOS, COmedication Study assessing Mono- and cOmbination therapy with levodopa-carbidopa inteStinal gel; CSAI, continuous subcutaneous apomorphine infusion; DA, dopamine; DUOGLOBE, DUOdopa/duopa in patients with advanced Parkinson’s disease-a GLobal OBservational study evaluating long-term Effectiveness; DYSCOVER, DYSkinesia COmparative interventional trial on duodopa VERsus Oral Medication; EQ-5D, European Quality of Life 5 Dimension; EQ-VAS, Euro Quality of Life Visual Analog Scale; GFQ, Gait and Falls Questionnaire; GLORIA, Global LOng-term Registry on efficacy and safety of LCIG in patients with Advanced Parkinson’s disease in routine care; GREENFIELD, Global REsponsE during iNFusIon of a gEl with LevoDopa/Carbidopa; LCIG, levodopa-carbidopa intestinal gel; MCSI, Modified Caregiver Strain Index; MAO-B, monoamine oxidase B; MMSE, Mini-Mental State Examination; NA, not assessed; NMDA, N-methyl-D-aspartate; NMSS, Nonmotor Symptoms Scale; PD, Parkinson’s disease; PDQ-8/39, 8-item/39-item Parkinson’s Disease Questionnaire; PDSS-2, Parkinson’s Disease Sleep Scale-2; QUIP-RS, Questionnaire for Impulsive-Compulsive Disorders in Parkinson’s Disease– Rating Scale; SD, standard deviation; UDysRS, Unified Dyskinesia Rating Scale; UPDRS, Unified Parkinson’s Disease Rating Scale; y, years. ^a^Data shown are from the 12-month interim analysis. ^b^Mean age of patients in treatment group. ^c^Time since onset of motor fluctuations. ^d^Measured during “Off” time. ^e^Remainder of patients managed on combination oral levodopa and decarboxylase inhibitor alone or in combination with COMT inhibitor.

**Table 2:** Motor symptom changes after initiation of infusion therapies

	LCIG/CLES							CSAI		
Motor Symptom	Olanow et al., 2014^a^ (NCT00357994 and NCT00660387) [20]	GLORIA [21]	COSMOS (NCT03362879) [22]	GREENFIELD [24]	DYSCOVER (NCT02799381) [23]	DUOGLOBE^b^ (NCT02611713) [25]	EuroInf (LCIG/CLES) [44]	TOLEDO (NCT02006121) [43]	EuroInf [44]	Borgemeester et al., 2016 [46]	Sesar et al., 2017 [47]
“Off” time – UPDRS IV item 39	NA	Improved	NA	Improved^c^	NA	NA	NA	NA	NA	NA	NA
“Off” time – patient’s diary	Improved^a^	NA	Improved	NA	Improved	Improved (patient report)	NA	Improved	NA	Improved	Improved
“On” time with dyskinesia – UPDRS IV item 32	NA	Improved	Improved	Improved^d^	NA	NA	NA	NA	NA	NA	NA
“On” time without troublesome dyskinesia – patient’s diary	Improved^a^	NA	NA	NA	Improved	NA	NA	Improved	NA	Improved	NA
“On” time resting tremor – UPDRS III item 20	NA	NA	NA	NA	NA	Improved	NA	NA	NA	NA	NA
“On” time action tremor – UPDRS III item 21	NA	NA	NA	NA	NA	Improved^e^	NA	NA	NA	NA	NA
UPDRS Part IV total score	NA	NA	Improved^f,g^	Improved^c^	NA	NA	Improved	NA	Improved	NA	NA
Dyskinesia severity – UPDRS IV item 33	NA	Improved^h^	NA	Improved^d^	NA	Improved	NA	NA	NA	NA	NA
Dyskinesia -related pain – UPDRS IV item 34	NA	Improved^h^	NA	Improved^d^	NA	Improved	NA	NA	NA	NA	NA
Early morning dystonia – UPDRS IV item 35	NA	Improved	NA	Improved^a^	NA	Improved	NA	NA	NA	NA	NA
UPDRS Part III	NS^c^	Improved^h^	NS	NA	Improved	Improved	Improved	NA	Improved	NA	Improved^i^
UDysRS	NA	NA	NA	NA	Improved	Improved	NA	NA	NA	NA	NA
UDysRS, Part I	NA	NA	NA	NA	Improved	Improved	NA	NA	NA	NA	NA
UDysRS, Part II	NA	NA	NA	NA	NA	Improved	NA	NA	NA	NA	NA
UDysRS, Part III	NA	NA	NA	NA	Improved	NS^j^	NA	NA	NA	NA	NA
UdysRS, Part IV	NA	NA	NA	NA	NA	Improved	NA	NA	NA	NA	NA
UDysRS, Historical score	NA	NA	NA	NA	Historical	Improved	NA	NA	NA	NA	NA
UDysRS, Objective score	NA	NA	NA	NA	NA	Improved	NA	NA	NA	NA	NA

This review does not include all studies published for each infusion therapy. CLES, carbidopa-levodopa enteral suspension; COSMOS, COmedication Study assessing Mono- and cOmbination therapy with levodopa-carbidopa inteStinal gel; CSAI, continuous subcutaneous apomorphine infusion; DUOGLOBE, DUOdopa/duopa in patients with advanced Parkinson’s disease-a GLobal OBservational study evaluating long-term Effectiveness; DYSCOVER, DYSkinesia COmparative interventional trial on duodopa VERsus Oral Medication; GLORIA, Global LOng-term Registry on efficacy and safety of LCIG in patients with Advanced Parkinson’s disease in routine care; GREENFIELD, Global REsponsE during iNFusIon of a gEl with LevoDopa/Carbidopa; LCIG, levodopa-carbidopa intestinal gel; NA, not assessed; NS, not significant; UDysRS, Unified Dyskinesia Rating Scale; UPDRS, Unified Parkinson’s Disease Rating Scale. ^a^Improvement versus oral immediate release levodopa/carbidopa. ^b^Data shown are from the 12-month interim analysis. ^c^Improvement at visits 2 and 3. ^d^Improvement at visit 3. ^e^Improvement at months 3, 6, and 12. ^f^Across all groups. ^g^LCIG monotherapy group showed greater improvement in nocturnal/morning akinesia versus LCIG daytime monotherapy and freezing of gait versus LCIG polytherapy. ^h^Improvement at last visit. ^i^Significant improvement when correcting the comparison for CSAI intervention duration. ^j^Improvement at day 1 and month 3 but not at months 6 and 12.

**Table 3:** Non-motor symptom and quality-of-life changes after initiation of infusion therapies

	LCIG/CLES							CSAI			
Scale	Olanow et al., 2014 ^a^ (NCT00357994 and NCT00660387) [20]	GLORIA [21]	COSMOS (NCT03362879) [22]	GREENFIELD [24]	DYSCOVER (NCT02799381) [23]	DUOGLOBE^b^ (NCT02611713) [25]	EuroInf (LCIG/CLES) [44]	TOLEDO (NCT02006121) [43]	EuroInf [44]	Borgemeester et al., 2016 [46]	Sesar et al., 2017 [47]
NMSS total score	NA	Improved	NS	NA	NA	Improved	Improved	NA	Improved	NA	NA
PDSS-2	NA	NA	NS	Improved	NA	Improved	NA	NA	NA	NA	NA
ESS total score	NA	NA	NA	NA	NA	Improved	NA	NA	NA	NA	NA
GFQ	NA	NA	NA	Improved	NA	NA	NA	NA	NA	NA	NA
QUIP-RS	NA	NA	NS	Improved	NA	NA	NA	NA	NA	NA	NA
PGIC	NA	NA	NA	NA	NA	NA	NA	Improved	NA	NA	NA
CGI-I	Improved	NA	NA	NA	Improved	NA	NA	NA	NA	Improved	NA
PDQ-39	Improved	NA	NA	Improved^c^	NA	NA	NA	NA	NA	NA	NA
PDQ-8	NA	Improved	NS	NA	Improved	Improved	Improved	NS^d^	Improved	NA	NA
EQ-5D	NS	Improved	NA	NA	NA	NA	NA	NA	NA	NA	NA
EQ-VAS	NA	Improved	NA	NA	NA	NA	NA	NA	NA	NA	NA
UPDRS Part I	NA	NA	NA	Improved^c^	NA	NA	NA	NA	NA	NA	NA
UPDRS Part II	Improved	Improved^e^	NA	Improved^c^	Improved	NS	NA	NA	NA	NA	Improved
MCSI	NA	NA	NA	NA	NA	Improved	NA	NA	NA	NA	NA

This review did not include all studies published for each infusion therapy. CGI-I, Clinical Global Impression-Improvement; CLES, carbidopa-levodopa enteral suspension; COSMOS, COmedication Study assessing Mono- and cOmbination therapy with levodopa-carbidopa inteStinal gel; CSAI, continuous subcutaneous apomorphine infusion; DUOGLOBE, DUOdopa/duopa in patients with advanced Parkinson’s disease-a GLobal OBservational study evaluating long-term Effectiveness; DYSCOVER, DYSkinesia COmparative interventional trial on duodopa VERsus Oral Medication; EQ-5D, European Quality of Life 5 Dimension; EQ-VAS, Euro Quality of Life Visual Analog Scale; ESS, Epworth Sleepiness Scale; GFQ, Gait and Falls Questionnaire; GLORIA, Global LOng-term Registry on efficacy and safety of LCIG in patients with Advanced Parkinson’s disease in routine care; GREENFIELD, Global REsponsE during iNFusIon of a gEl with LevoDopa/Carbidopa; LCIG, levodopa-carbidopa intestinal gel; MCSI, Modified Caregiver Strain Index; NA, not assessed; NMSS, Nonmotor Symptoms Scale; NS, not significant; PDSS-2, Parkinson’s Disease Sleep Scale-2; PDQ-8/39, 8-item/39-item Parkinson’s Disease Questionnaire; PGIC, Patient Global Impression of Change; QUIP-RS, Questionnaire for Impulsive-Compulsive Disorders in Parkinson’s Disease– Rating Scale, UPDRS, Unified Parkinson’s Disease Rating Scale. ^a^Improvement versus oral immediate release levodopa/carbidopa. ^b^Data shown are from 12-month interim analysis. ^c^Visit 2 and 3. ^d^Improved from day 1 to month 12. ^e^NS between groups.

**Table 4:** Most frequent adverse events (≥5%)

	LCIG/CLES						CSAI			
Study	Olanow et al., 2014 (NCT00357994 and NCT00660387) [20]	GLORIA [21]	COSMOS (NCT03362879) [22]	GREENFIELD [24]	DYSCOVER (NCT02799381) [23]	DUOGLOBE^a^ (NCT02611713) [25]	TOLEDO (NCT02006121) [40, 43]	EuroInf [44]	Borgemeester et al., 2016 [46]	Sesar et al., 2017 [47]
Most frequent AEs	Complication of device insertion, device-related complication, abdominal pain, procedural pain, constipation, nausea, flatulence, orthostatic hypotension^b^	Weight decreased, device-related infection^c^	— ^c^	— ^c^	Falling, procedural pain, depression, abdominal pain, anxiety, drug withdrawal syndrome, hypertension, urinary tract infection^d^	— ^e^	Skin reaction, infusion site nodules, nausea, somnolence, dyskinesia, fall, insomnia, constipation, dizziness, infusion site erythema, headache	Subcutaneous/local site discomfort, persisting nausea, severe somnolence^f^	Subcutaneous nodules, visual hallucination, peripheral edema, orthostatic hypotension, nausea or vomiting, hyperventilation	Subcutaneous nodules, orthostatic hypotension, nausea

This review did not include all studies published for each infusion therapy. AE, adverse event; CLES, carbidopa-levodopa enteral suspension; COSMOS, Comedication Study assessing Mono- and combination therapy with levodopa-carbidopa intestinal gel; CSAI, continuous subcutaneous apomorphine infusion; DUOGLOBE, DUOdopa/duopa in patients with advanced Parkinson’s disease-a Global Observational study evaluating long-term Effectiveness; DYSCOVER, DYSkinesia Comparative interventional trial on duodopa VERsus Oral Medication; GLORIA, Global Long-term Registry on efficacy and safety of LCIG in patients with Advanced Parkinson’s disease in routine care; GREENFIELD, Global ResponsE during iNFusIon of a gEl with LevoDopa/Carbidopa; LCIG, levodopa carbidopa intestinal gel; MedDRA, Medical Dictionary for Regulatory Activities; PD, Parkinson’s disease; SAE, serious adverse event. ^a^Data shown are from 12-month interim analysis. ^b^Most were mild to moderate in severity, all resolved, and occurred almost exclusively within the first week. ^c^Most frequent AEs occurred in≤5% of patients. ^d^Most AEs were rated mild or moderate in severity by investigators. ^e^11.8% (*n* = 23) patients had treatment-emergent serious AE with reasonable possibility of causal relationship with LCIG; however, treatment-emergent SAE for each MedDRA preferred term occurred in <  2% of patients. ^f^Most commonly reported side effects at 6 months of follow up.

Findings from multiple clinical and real-world studies have shown a substantial improvement in motor symptoms and non-motor symptoms in patients treated with LCIG/CLES (Tables 2 and 3) [21, 22, 24, 25]. Treatment with LCIG/CLES significantly (*p* <  0.001) improves dyskinesia assessed using Unified Dyskinesia Rating Scale (UDysRS) [23, 25], dyskinesia severity, dyskinesia-related pain, early morning dystonia, ADL, and QoL scores (Tables 2 and 3) [21, 22, 24, 25]. To our knowledge, the aforementioned DYSCOVER study is the only randomized clinical trial designed to investigate the efficacy of LCIG on dyskinesia as measured by the UDysRS [23]. Non-motor symptoms across many functional domains— cardiovascular, sleep/fatigue, mood/cognition, perceptual problems/hallucinations, attention/memory, gastrointestinal tract, urinary function, sexual function, and miscellaneous— also improve in response to LCIG/CLES treatment (Table 3) [1, 21, 22, 25].

Data from the COmedication Study assessing Mono- and cOmbination therapy with levodopa-carbidopa inteStinal gel (COSMOS) observational study showed that the proportion of patients with aPD treated with LCIG/CLES monotherapy increased from 15.2% (54/356) at treatment initiation to 31.7% (120/378) at month 12; LCIG/CLES daytime monotherapy increased from 13.2% (47/356) at initiation to 24.9% (94/378) at 12 months [21, 22]. A *post-hoc* analysis of data from the Global LOng-term Registry on efficacy and safety of LCIG in patients with advanced PD in routine care (GLORIA) showed statistically significant improvements of ADL, motor symptoms, “On” time with dyskinesia, and non-motor symptoms in patients receiving LCIG/CLES monotherapy compared to baseline of an overall similar magnitude as polytherapy [27].∥In the European Union, the product label states LCIG/CLES is usually administered during the patient’s awake period, and if medically justified, may be administered for up to 24 hours [18] to help reduce nocturnal symptomatic fluctuations and improve sleep [28]. Available evidence suggests that steady and continuous delivery of levodopa in patients treated with LCIG/CLES for 24 hours provides further improvement in motor and non-motor symptoms relative to the 16-hour infusion in certain patients [28]. Patients who may benefit from 24-hour LCIG/CLES infusion include those who present with adverse reactions to dopamine agonists, such as orthostatic hypotension and hallucinations, or those who experience severe “Off” time during the night [28]. In addition, 24-hour LCIG/CLES infusion may help improve rigidity and gait imbalances at night, reduce night falls, and increase “On” time in the early morning [28].∥A retrospective analysis on long-term safety and mortality of 79 patients treated with LCIG between 2005 and 2020 in Italy found a satisfactory long-term safety profile and efficacy, and a generally low rate of discontinuation (19 of 79 patients [24%]) [29]. The accompanying mortality analysis revealed a relatively long lifespan (median survival from disease onset, 25 years) in patients treated with LCIG, although this may not have been attributed to LCIG/CLES alone, as average follow-up with LCIG/CLES was approximately 47 months [29]. Although rare, patient death deemed probably related to treatment has occurred due to small bowel perforation and peritonitis [21] and intestinal obstruction [25].∥Infusion therapy with LCIG/CLES requires percutaneous gastrostomy, which carries inherent risks associated with any surgical procedure [30]. Real-world reports from patients have highlighted a few concerns related to the device or procedure, such as the size and weight of the pump, difficulty when dressing, special consideration when showering or swimming, the need to clean the tube daily, regular battery changes, and infections and itchiness at the stoma. In addition, traveling with the device was considered inconvenient, as cassettes require refrigeration. However, patients stated that the clinical benefits outweigh the side effects and complications related to the device or procedure [31].∥Infusion therapies that rely on intestinal pumps are also susceptible to gut microbial metabolism of levodopa, contributing to reduced bioavailability and higher dose requirements in some patients [32]. The most common adverse events with LCIG/CLES are listed in Table 4 and include device-related complications. In a small study (*N* = 103) in Slovenia, patients were followed up for ~14 years. The study results indicated that, although adverse events (AEs) were relatively common (severe dyskinesia: 32.0%, *n* = 33; higher probability of psychosis in older patients by Cox proportional hazards regression models: 1.06, *p* = 0.03), LCIG was effective in controlling motor complications and improving patients’ health-related quality of life [33].

### Other approved infusion therapy

Levodopa-entacapone-carbidopa intestinal gel (LECIG) infusion therapy is approved in some European countries and is also delivered via percutaneous endoscopic gastrostomy and percutaneous endoscopic gastro-jejunal tube over 16 hours. An important feature of LECIG therapy is the small size of the pump, which may be perceived as an important benefit; however, a smaller interface may also be challenging for certain patients or caregivers [31, 34, 35]. If necessary, physicians may opt to administer LECIG over 24 hours [36]. The inclusion of entacapone inhibits peripheral metabolism of levodopa, requiring a lower dose of LECIG to achieve similar levodopa exposure to that of LCIG/CLES [37]. Limited evidence is available regarding the efficacy and safety profile of LECIG, although the ELEGANCE study (NCT0504310) is an international, prospective, observational study that is underway in ~16 countries to collect real-world data on the efficacy and safety of LECIG.

### Clinical evidence for apomorphine

Another option available to treat patients with aPD whose symptoms are not sufficiently controlled with oral medication is continuous subcutaneous apomorphine infusion (CSAI), administered with a pump that is smaller and lighter than LCIG/CLES and does not require surgical placement [38, 39]. Apomorphine is a potent dopamine receptor agonist with antiparkinsonian efficacy [40]. Subcutaneous administration is necessary because oral apomorphine has poor bioavailability and a short half-life. Full 24-hour administration of apomorphine via CSAI is not advised to reduce the risk of tolerance to therapy [39, 41, 42]. Although 24-hour administration is possible in selected centers under expert monitoring, its wider use should not be advised. CSAI is not yet approved for use in the United States but the registration process is underway; it is approved in multiple other countries [38].

Evidence to support the efficacy of CSAI is derived from results from clinical trials; case reports; open-label, retrospective studies; case-control studies; and one pivotal, randomized, placebo-controlled trial (Table 1) [40, 43–47]. Results from both phases of the TOLEDO study showed that CSAI significantly (*p* <  0.01) reduced “Off” time and extended “On” time without troublesome dyskinesia (Table 2) [40, 43]. However, patients treated with CSAI did not report significant improvement in QoL at 12 weeks and 1 year, and issues with nausea, somnolence and nodules were noted with 24-hour administration (Table 3) [40, 43]. The effect of continuous apomorphine infusion on dyskinesia was only shown in a few small open label trials, which is insufficient evidence to propose this as a proven effective therapy in patients with dyskinesia [16, 48, 49]. Recently published Movement Disorder Society guidelines note that there is not sufficient controlled data to determine the effect of apomorphine on dyskinesias [16]. Similar findings were noted in a review on the efficacy of advanced treatments [49].

QoL was shown to improve with CSAI in the Euroinf Data study (Table 3), and significantly (*p* = 0.011) improved in 100 patients in the OPTIPUMP cohort study at 6 months [50]. Another study assessed add-on CSAI in 22 patients over a 6-month period and found a beneficial impact on QoL according to the SF-36 total score (*p* = 0.09) [51].∥Data from real-world and observational studies with interventions have shown CSAI efficacy in improving motor symptoms (Table 2) [44, 46, 47]. Long-term treatment with CSAI has also been associated with improved non-motor symptoms (Table 3) [44, 46].∥The main reasons patients discontinued CSAI therapy are presented in Table 4, and include lack of improvement of dyskinesia, hallucinations, skin nodules, and nausea [39, 40, 43, 44, 46, 47]. As CSAI involves subcutaneous administration, skin AEs at the injection site are common. However, skin reactions are typically mild or moderate in severity, and can often be managed without requiring treatment cessation [43].

## OVERVIEW OF EMERGING INFUSION THERAPIES

Additional infusion therapies such as once a week subcutaneous infusion of dopamine agonists or agents such as exenatide are under development and investigation while intracerebral infusion of trophic factors such as glial cell-derived neurotrophic factor (GDNF) and brain-derived neurotrophic factor (BDNF) have been examined.

Foslevodopa/foscarbidopa (previously known and referred to as ABBV-951) is a new soluble formulation of carbidopa and levodopa prodrugs delivered as a 24-hour/day continuous subcutaneous infusion by an infusion set connected to a portable pump [52–54]. On delivery to the body, foslevodopa/foscarbidopa is rapidly converted to the pharmacologically active levodopa/carbidopa via alkaline phosphatases, quickly reaching and maintaining steady-state therapeutic levels of plasma levodopa [52]. Foslevodopa/foscarbidopa has the potential to offer an efficacious and minimally invasive alternative therapy for patients with aPD and is undergoing approval by the FDA. Most AEs reported were non-serious and mild or moderate in severity [52–54]. Data from the pivotal study on the efficacy of 24-hour/day foslevodopa/foscarbidopa at week 12 revealed a significant (*p* = 0.0083) increase in hours of “On” time without troublesome dyskinesia and “Off” time compared to oral immediate-release levodopa-carbidopa [54].

ND0612 is a liquid formulation of levodopa-carbidopa delivered subcutaneously by a similar pump system as CSAI [55]. Patients may receive the infusion either continuously (24 hours) or during waking hours only [56]. ND0612 has an adequate safety profile and most patients treated with ND0612 have reported mild to moderate AEs [57]. Continuous administration of ND0612 elevated and sustained plasma levodopa to therapeutic levels, and 28-day intervention significantly improved motor symptoms from baseline to post-treatment in patients with aPD (“Off” time: *p* <  0.01; “On” time with dyskinesia: *p* <  0.001) [55, 56].

Infudopa is another soluble combination of levodopa and carbidopa that may be administered via intravenous injection, short-term intravenous infusion, and long-term subcutaneous infusion [58]. In a phase 1 clinical trial, patients presented with elevated and stable levels of plasma levodopa after a 16-hour subcutaneous infusion of infudopa [59]. Limited data on tolerability are available, but in the few patients who received subcutaneous infudopa infusion and presented with AEs, these events were mild to moderate [59, 60].

## IDENTIFYING PATIENTS WITH ADVANCED PARKINSON’S DISEASE WHO MAY BENEFIT FROM INFUSION THERAPY

A large multicenter observational study— OBSERVE-PD (OBSERVational, cross-sEctional PD)— acquired data from 2615 patients with PD in 18 countries and reported that only 43.6% of patients who met the criteria for aPD and were eligible for advanced treatment, were actually receiving it [61]. Although timely intervention with advanced treatment may result in better outcomes and improved QoL [13, 62, 63], international consensus on definitive diagnostic criteria for aPD and identification of patients who may benefit from advanced treatment is an unmet need in clinical practice [61, 64].

Some progress has been made on defining aPD. In 2018, a Delphi-panel approach was utilized to gain consensus from 17 leading movement disorders specialists on 15 clinical indicators for suspected aPD and seven eligibility criteria for device-aided therapies, considering motor and non-motor symptoms and functional impairments [62]. A retrospective analysis of the clinical indicators of aPD and device-aided therapy eligibility found all indicators demonstrated high clinical screening accuracy for identifying patients with aPD and determining those who were eligible to receive advanced treatment [65]. Further, OBSERVE-PD reported a moderate correlation (*K* = 0.430; 95% confidence interval 0.406–0.473) between physician’s judgment and the 2018 consensus criteria in 2615 patients [61]. Specific indicators of aPD included taking at least five oral levodopa doses per day, at least 2 hours of “Off” time per day, and at least 1 hour per day with troublesome dyskinesia, giving rise to the “5-2-1” criteria [62, 66]. *A post-hoc* analysis of data from a multicountry observational study of patients (*N* = 4714) with physician-identified aPD found that 33% of patients met the 5-2-1 screening criteria, with greater than 75% concordance between clinician judgment and the 5-2-1 criteria [66].

The MANAGE-PD (Making Informed Decision to Aid Timely Management of Parkinson’s Disease) tool, based on the Delphi consensus [62], is another instrument to help physicians identify patients who are not receiving adequate symptom control and may benefit from advanced treatment [67]. MANAGE-PD accounts for the impact of motor symptoms and non-motor symptoms and classifies patients into three categories regarding their current treatment regimen: 1) controlled with current therapy, 2) inadequately controlled with current therapy but may benefit from non– device-aided optimization, and 3) inadequately controlled with current treatment but may benefit from device-aided therapy [67]. The agreement between MANAGE-PD outcomes and ratings from PD specialists (as assessed by intra-class correlation coefficient) suggests that MANAGE-PD is a valid tool and complements physicians’ decision-making related to treatment optimization [67].

## CONSIDERATIONS AROUND OPTIMAL USE OF INFUSION THERAPY

Key areas for a successful infusion therapy include effective patient education; meticulous patient care and comprehensive assessments; interprofessional communication; and collaboration between patients, their caregivers, and knowledgeable and competent nurses [68].

While proper timing of infusion therapy is crucial for aPD treatment success, treatment also poses a challenge because of the numerous factors that influence the choice of therapy— including local availability and the differences among treatment centers, ranging from clinical experience to reimbursement [69]. The general recommendation is to initiate therapy before major disability occurs. For this reason, it is advised to start discussing advanced treatment with patients early in the disease course, preferably when motor fluctuations start to occur but are still manageable with modifications of oral dopaminergic therapy [69].

As initiation of infusion therapy is a highly individualized decision and often requires joint decision-making, patients and their caregivers are encouraged to actively participate in the process. Patient participation in decision-making concerning therapy positively correlates with patient satisfaction and adherence, and contributes to positive outcomes [70]. Joint decision-making involves assessing patients’ and their families’ expectations for the treatment, educating them about realistic treatment outcomes and symptoms that are more likely to improve, answering their questions, and giving the patient an active role in the process [70]. Unfortunately, many patients with aPD are reluctant to change their therapy regimen despite fulfilling the criteria for “advanced” therapies [61]. In one study nearly 38% of patients eligible for device-aided treatments reported no plan to use it, citing “needing more time to decide” (39%) and “refusal” (25%) as the two main reasons the eligible patients did not plan for advanced therapy [61]. A consensus guidance document was published on the “Navigate PD” pan-European educational program, which was developed to supplement existing guidelines, and provides insight on the many nuances of infusion therapy and patient management [13]. Considering emerging infusion therapies in the near future, treatment options for patients with aPD may expand to efficacious non-surgical subcutaneous therapy for controlling fluctuations [54].

Education on the most frequent AEs and common reasons for discontinuing each therapeutic option are also important aspects for patients and caregivers to consider in the decision-making process (Table 4) [70]. Patients and their families should be educated on caregiver burden and the resources that are available to them [71–73]. The lack of a caregiver may represent a challenge for patients considering infusion therapy, although this is somewhat dependent on local care facilities and services available [69, 74].

Multidisciplinary teams that include the treating neurologist, a PD nurse specialist, geriatrician, physical therapist, occupational therapist, speech therapist, nutritionist, and psychologist may improve treatment success, enhance QoL and ADL, provide strategies to support mental health, and ensure optimal nutrition [75, 76]. Positive outcomes require the commitment of all parties involved— patient, care provider, treating neurologist, and PD nurse specialist— who are crucial in complementing the therapeutic efforts of the rest of the team [70, 75]. PD nurse specialists play a key role in assisting patients and caregivers with their needs, as well as providing individual support, education, access to services, and coordination of care [77]. In this regard, PD nurse specialists can be a bridge of communication between the treating neurologist and the patient, helping improve therapy adherence [78]. By supporting individuals throughout all disease stages, PD nurse specialists can also contribute to the timely initiation of advanced care planning discussions, resulting in increased treatment initiation and reduced treatment discontinuations [70, 79]. Use of telemedicine may facilitate the communication between medical providers and patients, reduce commute time to attend in-person visits, and facilitate the implementation of home-based multidisciplinary care [80].

The literature on aftercare programs for patients with PD, and their influence on long-term adherence to infusion therapy, and on caregivers’ burden, is scarce. Studies have analyzed the reasons patients discontinue infusion therapies and found important factors that can increase therapeutic adherence and long-term benefits of infusion therapy. These include clinician experience in selecting and treating patients with aPD, the presence of a well-trained caregiver, and the availability of a proper aftercare system [81–83].

## CONCLUSIONS

This review summarizes the benefits of infusion therapy in patients with aPD and outlines considerations around its optimal use. Although a lack of consensus around the identification of aPD represents an ongoing challenge, and advanced treatments are underused in aPD, new tools or simple criteria have been proposed to assist clinicians in advanced disease staging and patient classification for infusion therapy eligibility. Several studies show that patient and physician acceptance of infusion therapies may become less of a barrier with appropriate multidisciplinary management and proper patient education, especially with the key contributions made by PD nurse specialists.
